# A rapidly enlarging thyroid mass

**DOI:** 10.11604/pamj.2022.42.112.32997

**Published:** 2022-06-10

**Authors:** Georgios Meristoudis, Ioannis Ilias

**Affiliations:** 1 Department of Nuclear Medicine, Hippokration General Hospital, Thessaloniki, Greece,; 2 Department of Endocrinology, Diabetes and Metabolism, Elena Venizelou Hospital, Athens, Greece

**Keywords:** Primary thyroid lymphoma, scintigraphy, gallium-67, technetium-99m

## Image in medicine

We report the case of a 67-year-old woman who presented with a six-week history of a neck mass, mild dysphagia and dyspnea, which were accompanied by night sweats and low-grade fever. On physical examination, the thyroid gland was hard/non-tender on palpation and diffusely and asymmetrically enlarged (especially to the right). Laboratory evaluation revealed a thyroid-stimulating hormone level of 9.97 mIU/L (normal range: 0.38-5.33), a free thyroxine level of 0.6 ng/dL (normal range: 0.6-1.49) and thyroid peroxidase antibody titer > 600 IU/mL (normal < 50). Technetium-99m thyroid scintigraphy showed decreased radiotracer uptake throughout the thyroid (A). The laboratory test results and scintigraphic findings were suggestive of Hashimoto´s thyroiditis. Nevertheless, a core biopsy of the thyroid mass was done, due to the clinical suspicion of malignancy, revealing a diffuse large B-cell lymphoma. Subsequent gallium-67 scintigraphy demonstrated abnormal intense uptake only in the region of the thyroid mass (B, C). The thoracic and abdominal computerized tomographies were normal. The patient was treated with chemotherapy (rituximab plus cyclophosphamide, doxorubicin, vincristine, and prednisone), followed by radiotherapy. Six months after treatment, she is in good health. Primary thyroid lymphoma (PTL) is rare: it represents less than 5% of all thyroid malignancies and less than 3% of extra-nodal lymphomas. Differentiation of PTL from other thyroid neoplasias is necessary; it is treated differently and has a relatively good prognosis. The differential diagnosis of PTL includes goitrous Hashimoto´s thyroiditis, anaplastic thyroid carcinoma or other infiltrative thyroid neoplasms.

**Figure 1:**
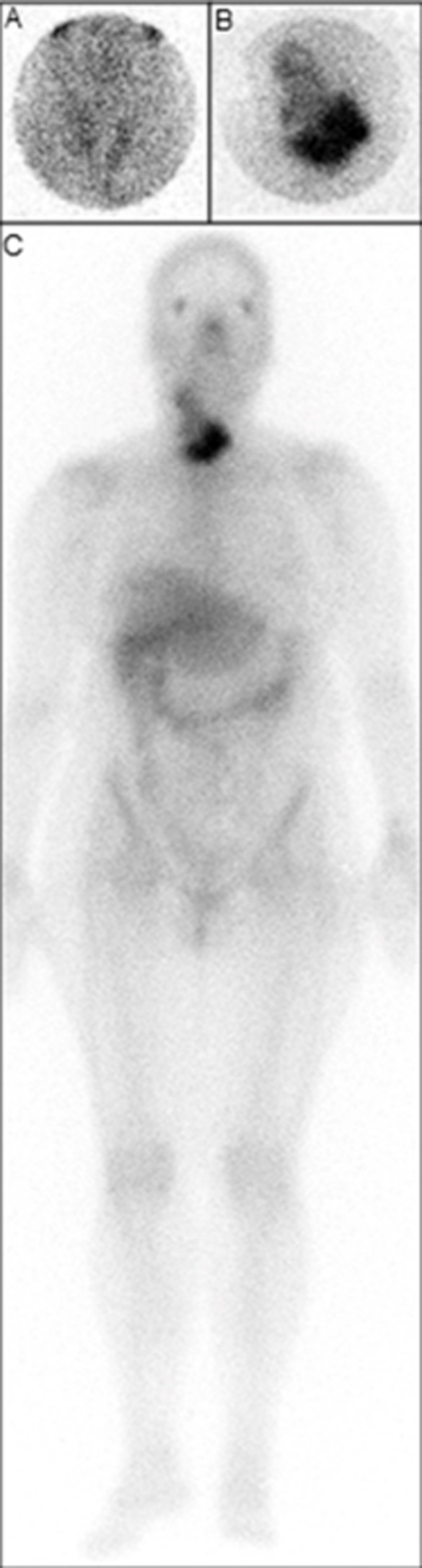
anterior-pinhole (magnified) images of the neck; A) technetium-99m scintigraphy, showing decreased thyroid uptake; B) gallium-67 scintigraphy, demonstrating marked accumulation in the thyroid lymphoma; C) whole-body gallium-67 scintigraphy

